# α-Synuclein and DJ-1 as Potential Biological Fluid Biomarkers for Parkinson’s Disease

**DOI:** 10.3390/ijms11114257

**Published:** 2010-10-29

**Authors:** Masaaki Waragai, Kazunari Sekiyama, Akio Sekigawa, Yoshiki Takamatsu, Masayo Fujita, Makoto Hashimoto

**Affiliations:** Laboratory for Chemistry and Metabolism, Tokyo Metropolitan Institute for Neuroscience, Tokyo, 183-8526, Japan; E-Mails: sekiyama-kz@igakuken.or.jp (K.S.); sekigawa-ak@igakuken.or.jp (A.S.); takamatsu-ys@igakuken.or.jp (Y.T.); fujita-ms@igakuken.or.jp (M.F.); hashimoto-mk@igakuken.or.jp (M.H.)

**Keywords:** Parkinson’s disease, cerebrospinal fluid, biomarker, α-synuclein, DJ-1

## Abstract

Parkinson’s disease (PD) is the most common form of movement disorder and affects approximately 4% of the population aged over 80 years old. Currently, PD cannot be prevented or cured, and no single diagnostic biomarkers are available. Notably, recent studies suggest that two familial PD-linked molecules, α-synuclein and DJ-1, are present in cerebrospinal fluid (CSF) and that their levels may be altered during the progression of PD. In this regard, sensitive and accurate methods for evaluation of α-synuclein and DJ-1 levels in the CSF and blood have been developed, and the results suggest that the levels of both molecules are significantly decreased in the CSF in patients with PD compared with age-matched controls. Furthermore, specific detection and quantification of neurotoxic oligometric forms of α-synuclein in the blood using enzyme-linked immunosorbent assays might be expected as potential peripheral biomarkers for PD, although further validation is required. Currently, neither α-synuclein nor DJ-1 is satisfactory as a single biomarker for PD, but combinatory evaluation of these biological fluid molecules with other biomarkers and imaging techniques may provide reliable information for diagnosis of PD.

## 1. Introduction

Biological fluid biomarkers in blood samples, urine and cerebrospinal fluid (CSF) are measurable markers of underlying disease. For example, plasma prostate-specific antigen has been established as a biomarker for screening, early diagnosis, and tracking of tumor progression in prostate cancer [1]. Blood hemoglobin A1c is commonly used for diagnosis of diabetes mellitus and for predicting and monitoring the severity of cardiovascular events [2]. In neurodegenerative diseases such as Alzheimer’s disease (AD), alterations of amyloidogenic proteins, including β-amyloid and tau, have been widely investigated. The levels of these molecules in the CSF are insufficient for them to be utilized as biomarkers, but the recent AD Neuroimaging Initiative study indicated that CSF β-amyloid and tau protein analysis combined with neuroimaging techniques such as magnetic resonance imaging (MRI) and positron emission tomography (PET) may provide reliable information for early diagnosis and tracking of severity in AD [3]. Advance of neuroimaging methods, such as a high field MRI showing increased iron content in the substantia nigra [4,5], [^123^I]-meta-iodobenzylguanidine myocardial scintigraphy [6], and PET [7], could be also useful for the diagnosis of early stages of Parkinson’s disease (PD). However, these examinations are generally expensive and are not available in all hospitals. Thus, much expectation has been paid to the discovery of simple and convenient biomarkers specific for the pathogenesis of PD.

PD is a common neurodegenerative disease that presents with tremor, rigidity and postural instability. PD is characterized pathologically by selective degeneration of the dopaminergic neurons of the substantia nigra in association with formation of Lewy body inclusions and Lewy dystrophic neuritis [8]. Since the discovery of missense mutations (A53T and A30P) of α-synuclein in familial cases of PD (PARK1), numerous histological studies have shown that α-synuclein is a major component in Lewy bodies [8]. Subsequently, several familial PD-linked mutations, including parkin (PARK2), UCHL-1 (PARK5), PINK1 (PARK6), DJ-1 (PARK7), LRRK2 (PARK8) and ATP13A2 (PARK9), have been identified [9]. For α-synuclein, gene triplication has been found in familial PD (PARK4) [9] and an E46K mutation was identified in a family with dementia with Lewy bodies (DLB) [10].

Given the common neuropathological features between familial and sporadic cases of PD, changes in familial PD-linked molecules are likely to be involved in the pathogenesis of sporadic PD. Thus, these PD-linked molecules are candidate biomarkers for PD. In this context, several studies have compared the levels of CSF and blood α-synuclein and DJ-1 between PD patients and non-PD controls. Although the results are conflicting, it appears that α-synuclein and DJ-1 in CSF, but not in blood, are significantly decreased in PD patients compared to non-PD controls [11,12]. Therefore, the purpose of this review is to summarize the studies on CSF and blood α-synuclein and DJ-1 as possible biomarkers for PD.

## 2. Evaluation of CSF and Blood α-Synuclein as a Biomarker of PD

Previous studies have shown that concentrations of CSF biomarkers for AD, such as β-amyloid and tau, correspond well with brain alterations during disease progression [13]. In a similar context, several studies have evaluated CSF α-synuclein levels in PD. Jakowec *et al.* performed immunoblot analysis, but failed to detect the native form of α-synuclein in the CSF of PD patients and non-PD controls [14]. Borghi *et al.* identified a 19 kDa monomeric α-synuclein by immunoprecipitation and immunoblotting with different anti-α-synuclein antibodies, and concluded that the amount of CSF α-synuclein did not vary significantly between PD and non-PD cases [15]. Ohrfelt *et al.* measured CSF α-synuclein levels with enzyme-linked immunosorbent assays (ELISA) that allows for precise quantification (down to 50 pg/mL) of α-synuclein in CSF, but failed to see any significant differences among PD, DLB and their controls [16]. Thus, these studies suggested that α-synuclein in CSF is not a useful biomarker of PD. In contrast, Mollenhauer *et al.* and Tokuda *et al.* performed ELISA to measure CSF α-synuclein levels and found significant decreases of α-synuclein in PD patients compared with non-PD controls [17,18]. Interestingly, Tokuda *et al.* observed a strong inverse correlation between CSF α-synuclein levels and disease severity evaluated by Hoenh-Yahr stage, and suggested that the CSF α-synuclein levels may be a potential marker to aid clinical diagnosis of PD [18]. Thus, these studies have yielded conflicting results.

Characterization of α-synuclein in blood has also been performed by several groups, and again the results are conflicting. Lee *et al.* measured the blood α-synuclein levels using a commercially available ELISA kit in 105 PD patients, 38 MSA patients, and 51 age-matched controls. The α-synuclein level was significantly elevated in patients with PD and MSA compared with controls, and was higher in patients with PD compared to those with MSA [19]. In contrast, Li *et al*. performed semi-quantitative immunoblot analysis to measure the blood α-synuclein levels using antibody 97/8, which is specific for the α-synuclein *C*-terminal region, in 27 PD and 11 non-PD cases, and found significantly decreased α-synuclein levels in the PD cases compared to the non-PD controls [20].

Several factors may account for the conflicting results, including variations in detection, sensitivity and accuracy in each experimental system. It is also possible that the different antibodies used in different assays might detect different species of the proteins. As Li *et al.* explained, the advantage of the Western blotting method is that it can measure specific species of full-length α-synuclein, while ELISA systems might detect truncated α-synuclein in addition to the full-length protein, as well as oligomers and other cross-reactive molecules [20]. In addition, blood contamination may prevent accurate evaluation of the CSF α-synuclein level, since Nakai *et al.* showed that more than 90% of peripheral erythrocytes expressed α-synuclein [21] and it has been shown that α-synuclein is abundantly expressed in platelets [22]. Thus, CSF or plasma α-synuclein levels are likely to be increased when blood contamination or possible platelet stimulation occurs during sample preparation.

The problem of blood contamination was conquered by Hong *et al.* who studied CSF α-synuclein levels using sensitive and quantitative Luminex bead-based assays in large numbers of subjects (117 PD patients, 132 healthy individuals, and 50 AD patients) [11]. To address the possibility of blood contamination, a hemoglobin ELISA was performed for all CSF samples [11]. Notably, the CSF α-synuclein and DJ-1 levels were substantially increased in all samples that were contaminated with blood (>200 ng/mL hemoglobin). Thus, this study elegantly demonstrated that CSF α-synuclein levels are decreased in PD cases compared to non-PD controls and AD cases, although there was no correlation with the severity of the disease.

Another important issue in evaluation of CSF α-synuclein is that the protein is prone to aggregation. Oligomeric and protofibril forms may play a role in neurotoxicity, and these forms, rather than the monomer, may be appropriate targets for evaluation of CSF α-synuclein as a biomarker. Thus, El-Agnaf *et al.* developed a novel ELISA that detects exclusively oligomeric “soluble aggregates” of α-synuclein [23], and found that oligomeric forms of α-synuclein were significantly elevated in serum samples from PD patients compared with non-PD controls. This was the first demonstration that the extent of aggregation of α-synuclein is reflected in the serum of PD patients. However, since α-synuclein aggregates are composed of heterogeneous populations, it is unclear whether pathogenic aggregates are recognized by the anti-α-synuclein antibody, and further studies are required to address this question. Furthermore, since it is well established that Ser129-phosphorylation of α-synuclein is associated with aggregation of α-synuclein and Lewy body formation [24], it is important to determine whether phospho-α-synuclein can be used as a peripheral biomarker.

Finally, emerging evidence suggests a role of secretion and possible propagation of α-synuclein in the pathogenesis of PD [25]. The occurrence of Lewy pathology in neurons grafted into the brains of PD patients [26,27] suggests that α-synuclein pathology can spread from host tissues to the grafts. The mechanism underlying this propagation is still unclear. If such a prion-like transmissible form of α-synuclein is indeed isolated in CSF, it may then be an ideal biomarker that is directly correlated with disease progression. Thus, future studies of CSF α-synuclein may provide critical insights into the mechanism of pathological progression in PD and other proteinopathies.

## 3. Evaluation of CSF and Blood DJ-1 as a Biomarker of PD

DJ-1 was first identified by Nagakubo *et al*. as a novel oncogene that works in association with ras in cellular transformation [28]. It has subsequently been shown that DJ-1 is a multifunctional protein that is involved in various cellular processes, including response to oxidative stress, RNA binding, androgen-receptor signaling, spermatogenesis, and fertilization [29]. DJ-1 is secreted into the serum of patients with breast cancer, melanoma, familial amyloidotic polyneuropathy, and stroke [29]. Since the discovery of the linkage of gene mutations of DJ-1 to autosomal recessive familial PD (PARK7), much attention has been paid to the role of this molecule in the pathogenesis of PD and related neurodegenerative diseases [30]. Further investigation has shown that DJ-1 may play a protective role in oxidative stress during neurodegeneration [31].

Choi *et al.* reported that the total level of DJ-1 measured by semi-quantitative immunoblot analysis is significantly increased in PD and AD brains [32]. Consistent with this, we observed that both monomer and aggregates of DJ-1 were abundantly present in the CSF, and our semi-quantitative immunoblot analysis revealed that CSF-1 DJ-1 monomer levels were significantly increased in PD compared to those in non-PD controls [33]. We also found that plasma DJ-1 levels were increased in PD cases compared to non-PD controls [34]. Thus, our results raise the possibility that CSF or serum DJ-1 could be used as a possible biomarker for PD.

Our findings stimulated investigation of CSF and blood DJ-1 in the PD field, but the results obtained by other groups were variable and mostly contradictory to our data. Maita *et al.* examined DJ-1 levels using a DJ-1 ELISA kit in PD cases and age-matched controls, but did not find a significant difference in the levels of secreted DJ-1 between these groups or correlations of DJ-1 levels with age and clinical severity [35]. Hong *et al.* conducted a careful measurement of CSF DJ-1, as described above for CSF α-synuclein [11], with measurement of CSF DJ-1 levels using quantitative Western blotting, in-gel digestion and mass spectrometry, size-exclusion chromatography, and a newly developed and highly sensitive bead-based Luminex assay [11]. Hemoglobin levels in the CSF samples were measured to check for the effect of blood contamination [11]. The results showed that CSF DJ-1 and α-synuclein levels were dependent on age and influenced by the extent of blood contamination in the CSF. Both DJ-1 and α-synuclein levels were decreased in PD cases compared to non-PD controls and AD cases after elimination of the effect of blood contamination. However, there was no association between DJ-1 or α-synuclein and the severity of PD [11]. These findings led to the conclusion that total DJ-1 and α-synuclein in human CSF are helpful diagnostic markers for PD, if variables such as blood contamination and age are taken into consideration. This work provides a reliable evaluation of CSF α-synuclein and DJ-1 levels as biomarkers for PD. Subsequently, the same group showed that plasma DJ-1 or α-synuclein levels did not differ in PD cases compared to age-matched controls and AD cases, and concluded that, unlike in the CSF, total DJ-1 or α-synuclein in plasma alone is not useful as a biomarker for diagnosis, progression or severity of PD [12].

Finally, it is worth noting that Ooe *et al.* developed specific antibodies that recognize C106-oxidized DJ-1 [36]. Examination of the oxidized DJ-1 levels could be more meaningful because abnormal oxidized DJ-1 has been specifically increased in patients with PD and AD [32,37] and measurement of oxidized DJ-1 in the CSF or blood using these specific DJ-1 antibodies with Western blot and ELISA system could be useful.

## 4. Other Potential Blood Biomarkers for PD

At this stage, neither α-synuclein nor DJ-1 alone appears to be satisfactory as a single biological fluid biomarker for PD. However, combinatory analysis of these molecules with other data might give useful information. Indeed, previous works have investigated various molecules related to the pathogenesis of PD.

First, oxidative stress and mitochondrial respiratory failure are implicated in loss of dopaminergic neurons in PD. Alam *et al.* showed a selective increase in 8-hydroxydeoxyguanosine (8-OHdG), a product of oxidized DNA or RNA, in the substance nigra in PD [38]. Kikuchi *et al.* showed increased levels of 8-OHdG in the serum and CSF in patients with PD and multiple system atrophy, and suggested that systemic DNA/RNA oxidation is common in neurodegenerative diseases [39]. More recently, Sato and Hattori used an ELISA to show that urinary levels of 8-OHdG were increased in PD patients, and that mean urinary 8-OHdG levels increased with disease progression, which suggests that urinary 8-OHdG is a potential biomarker for progression of PD [40].

Second, neuroinflammatory processes are well known to be associated with the pathogenesis of neurodegenerative diseases, including PD [41]. Activated microglial cells are found within the substantia nigra in autopsied PD patients, and increased levels of proinflammatory cytokines such as tumor necrosis factor α (TNF-α), interleukin 6 (IL-6) and interleukin 1β (IL-1β) are also found in the substantia nigra in PD [42]. Inflammatory cytokines in biological fluids (CSF or serum) obtained from PD patients have been studied, since the levels of these molecules may reflect glial activation and inflammation in the brain [42]. Changes in the levels of proinflammatory molecules such as TNF-α, IL-6 and IL-1β, which may be derived from activated microglia, have been found in the CSF of PD patients.

Third, the neurodegenerative process of PD is associated with altered expression of growth factors. Godau *et al.* recently showed that the levels of serum insulin-like growth factor (IGF-1) were significantly higher in treated PD patients than in controls [43]. Interestingly, higher serum IGF-1 levels were correlated with shorter disease duration, and this led to the conclusion that increased IGF-1 might be a serum marker for early PD and potentially for subclinical dopaminergic dysfunction. However, the clinical value of this assay for diagnosis of PD is uncertain.

## 5. Conclusions

A number of recent studies have investigated α-synuclein and DJ-1in CSF and blood as potential biomarkers for PD. Among the conflicting results, a most sophisticated work evaluating the possibility of blood contamination suggests that both α-synuclein and DJ-1 in CSF may be decreased in PD compared to non-PD controls. Nonetheless, no correlation with the disease severity has been confirmed. Thus, these results indicate that neither α-synuclein nor DJ-1 is satisfactory as a single biological fluid biomarker for PD. In this situation, combinatory analysis of CSF α-synuclein and/or DJ-1 with other PD biomarkers, such as oxidative stress-related molecules, pro-inflammatory factors and some growth factors, might be effective. Alternatively, developing methods to detect specific molecules underlying PD pathogenesis, such as oligomer, protofibrils and putative transmissible form of α-synuclein taken from blood and CSF combined with genetic screenings of PD linked genes and imaging techniques would increase the possibility of detecting disease.
